# Editorial: Phytochemicals and therapeutic targets: their interactions and effects on health

**DOI:** 10.3389/fnut.2023.1269404

**Published:** 2023-08-23

**Authors:** Li Ren, Carlos L. Cespedes-Acuña, Jie Zhang

**Affiliations:** ^1^College of Food Science and Engineering, Jilin University, Changchun, China; ^2^Basic Sciences Department, University of Bio Bio, Chillan, Chile

**Keywords:** phytochemicals, therapeutic targets, interactions, chronic diseases, regulation mechanisms

As naturally occurring compounds with widespread distribution in various plants and fruits, phytochemicals exhibit multiple pharmacological activities including anti-microbial, anti-oxidative, anti-inflammatory, and anti-cancer activities (1, 2). Phytochemicals play a role in the nutritional intervention of chronic diseases via potential therapeutic targets, such as nuclear receptors and transmembrane receptors (3). Mechanistically, phytochemicals can bind to the respective target proteins and subsequently regulate their transcription. Thus, the interactions between phytochemicals and therapeutic targets should be investigated to explore the mechanisms for health benefits of phytochemicals. Since natural products offer significant advantages over synthetic drugs (4), screening of novel modulators of target proteins from phytochemicals may be a promising therapeutic approach for human diseases. This Research Topic aims to explore the interactions of phytochemicals with therapeutic targets, thus elucidating the regulation mechanisms of phytochemicals on human chronic diseases.

As a nutritional and nutraceutical resource for human and animal diets, *Cyclocarya paliurus* (*C. paliurus*) possesses multiple pharmacological activities such as antihypertensive, antioxidant, anticancer, antimicrobial, and immunological activities. Shen et al. summarized the nutritional composition of *C. paliurus*, including polysaccharides, triterpenoid saponins, polyphenols, and flavonoids. They also reviewed the research progress on the extraction methods, structural characteristics, and biological activities of these phytochemicals. Although *C. paliurus* is a promising candidate for developing functional ingredients in traditional Chinese medicine, a more profound understanding of its active compounds and action mechanisms is required.

Aberrations in stress signaling pathways, such as nuclear factor erythroid 2-related factor 2 (NRF2)/Kelch-like ECH-associated protein (KEAP1), may be linked to the development of lung adenocarcinoma. Datta et al. investigated the selective reactive oxygen species-dependent anticancer efficacy of the theaflavin-rich black tea (BT). Intriguingly, BT acted as a better anticancer agent than synthetic NRF2 modulators in the regulation of NRF2-KEAP1 and their upstream networks. Thus, as a potent and selective NRF2-modulator, BT might serve as a promising anticancer agent either as a single agent or in combination with other cancer chemotherapeutics.

As a common alcoholic liver disease, alcohol-induced acute liver injury (ALI) is one of the causes of liver failure and even liver cancer. Li et al. investigated the hepatoprotective effect of traditional Chinese medicine-probiotics complex (TCMPC) and its underlying mechanism for the treatment of ALI in mice. TCMPC reduced the level of liver injury biomarkers and also regulated oxidative stress, thereby ameliorating ALI in mice. Furthermore, it significantly reduced the production of inflammatory cytokines caused by ALI. This work might provide a new way for liver disease treatment by using phytochemicals.

In addition to the aforementioned experimental studies, Satomi et al. further explored the effects of glucoraphanin on the biological markers related to liver function in healthy individuals. They conducted a randomized, double-blind, placebo-controlled, parallel-group trial from April 22 to December 25, 2021. Glucoraphanin significantly improved serum alanine aminotransferase levels at 24 weeks compared to placebo supplements. In conclusion, daily intake of the glucoraphanin supplements might be beneficial for maintaining liver health in healthy, middle-aged adults with high-normal serum hepatic biomarkers.

In summary, these studies confirmed the health benefits of phytochemicals on human chronic diseases and explored their regulation mechanisms. Hence, the clinical applications of phytochemicals might be accelerated with the support of these experimental evidences and randomized trials.

## Author contributions

LR: Writing—original draft. CC-A: Writing—original draft. JZ: Writing—review and editing.
